# “Valencene” Produced Using *Rhodobacter Sphaeroides* 168 strain (Genetically Modified Foods and Feeds)

**DOI:** 10.14252/foodsafetyfscj.D-23-00003

**Published:** 2023-06-23

**Authors:** Food Safety Commission of Japan

## Abstract

Food Safety Commission of Japan (FSCJ) conducted a safety assessment on a food additive: flavoring “Valencene”, which is produced using *Rhodobacter sphaeroides* 168 strain based on documents mainly submitted by the applicant. Safety of the inserted genes including toxicity and allergenicity of the proteins produced from the inserted genes, recombinant and host protein residues, and others were evaluated based on the guideline. In the evaluations no risk due to use of recombinant technology was found in the bio-production of “Valencene”. From the identified chemical structures, toxicological findings and also estimated intakes of non-active ingredients detected in “Valencene”, none of safety issues were expected for them. From the above evaluations, FSCJ concluded that no concern relevant to human health is raised on the food additive, “Valencene” produced using *Rhodobacter sphaeroides* 168 strain.

## Conclusion in Brief

Food Safety Commission of Japan (FSCJ) conducted a safety assessment on a food additive: flavoring “Valencene”, which is produced using *Rhodobacter sphaeroides* 168 strain based on documents mainly submitted by the applicant.

*Rhodobacter sphaeroides* 168 strain was generated through the introduction of an expression vector: p-m-Pppa-MBP-ValC-mpmii alt into *Rhodobacter sphaeroides* 35053 strain as a host. The expression vector was constructed by insertion of a gene cluster involved in mevalonate syntheses derived from *Paracoccus zeaxanthinifaciens* ATCC 21588 strain, genes encoding isopentenyl diphosphate isomerase and other proteins derived from *Escherichia coli* DH5α, and a modified valencene synthase gene derived from *Callitropsis nootkatensis* into a plasmid: pBBR1MCS-2. “Valencene” is used as a flavor in food and drink such as juice and chewing gum, as the same way as the conventional Valencene (classified into terpene hydrocarbons listed in the Existing Food Additives of Japan) purified from orange.

Safety of the inserted genes including toxicity and allergenicity of the proteins produced from the inserted genes, recombinant and host protein residues, and others were evaluated based on the “Standards for Safety Assessments of Food Additives Produced Using Genetically Modified Microorganisms”*^1^. In the evaluations no risk due to use of recombinant technology was found in the bio-production of “Valencene”.

In addition, chemical analysis mainly using gas chromatography-mass spectrometry supported the higher specific content of valencene in the recombinant product than the conventional Valencene from orange. “Valencene” contained multiple new non-active ingredients and the higher existence of a non-active ingredient than the conventional Valencene. From identified chemical structures, toxicological findings and also estimated intakes of these non-active ingredients, none of safety issues were expected for them.

From the above, FSCJ judged that there is no factor bringing out adverse effects on humans in “Valencene”, compared with the conventional Valencene.

Hence, FSCJ concluded that no concern relevant to human health is raised on the food additive, “Valencene” produced using *Rhodobacter sphaeroides* 168 strain.
